# Dibenzazepine promotes cochlear supporting cell proliferation and hair cell regeneration in neonatal mice

**DOI:** 10.1111/cpr.12872

**Published:** 2020-07-17

**Authors:** Jingfang Wu, Xinran Dong, Wen Li, Liping Zhao, Li Zhou, Shan Sun, Huawei Li

**Affiliations:** ^1^ Otorhinolaryngology Department of Eye & ENT Hospital Fudan University Shanghai China; ^2^ NHC Key Laboratory of Hearing Medicine State Key Laboratory of Medical Neurobiology and MOE Frontiers Center for Brain Science Institutes of Biomedical Sciences Fudan University School of Basic Medical Sciences Shanghai China; ^3^ Molecular Medical Center Children's Hospital of Fudan University Shanghai China; ^4^ Shanghai High School Shanghai China; ^5^ The Institutes of Brain Science and the Collaborative Innovation Center for Brain Science Fudan University Shanghai China

**Keywords:** cell proliferation, cell regeneration, dibenzazepine, hair cells, Notch signal, supporting cells

## Abstract

**Objectives:**

To investigate the role of dibenzazepine (DBZ) in promoting supporting cell (SC) proliferation and hair cell (HC) regeneration in the inner ear.

**Materials and Methods:**

Postnatal day 1 wild‐type or neomycin‐damaged mouse cochleae were cultured with DBZ. Immunohistochemistry and scanning electron microscopy were used to examine the morphology of cochlear cells, and high‐throughput RNA‐sequencing was used to measure gene expression levels.

**Results:**

We found that DBZ promoted SC proliferation and HC regeneration in a dose‐dependent manner in both normal and damaged cochleae. In addition, most of the newly regenerated HCs induced by DBZ had visible and relatively mature stereocilia bundle structures. Finally, RNA sequencing detected the differentially expressed genes between DBZ treatment and controls, and interaction networks were constructed for the most highly differentially expressed genes.

**Conclusions:**

Our study demonstrates that DBZ can significantly promote SC proliferation and increase the number of mitotically regenerated HCs with relatively mature stereocilia bundles in the neonatal mouse cochlea by inhibiting Notch signalling and activating Wnt signalling, suggesting the DBZ might be a new therapeutic target for stimulating HC regeneration.

## Graphical abstract

Hair cell (HC) damage is the main cause of permanent hearing loss in mammals. To investigate the role of DBZ in the neonatal mouse cochlea, we cultured postnatal day 1 mouse cochleae with DBZ. We found that DBZ promoted SC proliferation and new HC generation in a dose‐dependent manner in both intact and damaged cochleae. In addition, most of the newly generated HCs induced by DBZ had visible and relatively mature stereocilia bundle structures. Our study demonstrates that DBZ can significantly promote SC proliferation and increase the number of mitotically regenerated HCs with relatively mature stereocilia bundles in the neonatal mouse cochlea by inhibiting Notch signalling and activating Wnt signalling, suggesting the DBZ might be a new therapeutic target for stimulating HC regeneration.

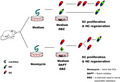

## 1 INTRODUCTION

Hair cells (HCs) in the cochlea are a critical part of the auditory system and convert the vibrations of sound waves into electrical signals. The HCs are vulnerable to various insults such as acoustic trauma, ageing, noise exposure and ototoxic drugs. When mammalian cochlear HCs are damaged, this leads to permanent hearing loss due to the inability to regenerate new HCs spontaneously after the cochlear HCs have differentiated and matured.^1^ However, the lower vertebrates, like fish and birds, can regenerate new HCs spontaneously when the cochlear HCs are damaged, and it has been shown that the mammalian utricle has very limited spontaneous HC regeneration ability.^2^, ^3^, ^4^

Recent studies have shown that supporting cells (SCs) are reliable sources for regenerating HCs after HC loss in both the cochlea and the utricle. There are two kinds of HC regeneration mechanisms in vertebrates, namely direct transdifferentiation, in which SCs directly differentiate into HCs without cell division,^5^, ^6^ and mitotic regeneration, in which SC/progenitor cells re‐enter the cell cycle to proliferate first and then, several days later, switch fates to become HCs.^7^, ^8^ In the mouse cochlea, HCs are interdigitated by SCs, and previous studies have shown that SC loss also leads to apoptosis of HCs.^9^ Thus, the goal of HC regeneration strategies is to regenerate the HCs without exhausting the SCs at the same time, and in our work we aim to induce SC/progenitor cell proliferation first and then induce the proliferated SCs/progenitor cells to differentiate into HCs.

Dibenzazepines (DBZs), which are a class of drugs that includes clozapine, carbamazepine and many others, is mainly used for the treatment of epilepsy, trigeminal neuralgia and some psychiatric disorders. All of these drugs are heterocyclic compounds consisting of two benzene rings fused to an azepine ring. Recently, DBZ (also known as γ‐secretase inhibitor XX (GSI XX)), a member of the dibenzazepine family, has shown effects on Notch signalling pathways,^10^ and the use of DBZ has been extended to other diseases due to its role in modulating Notch signalling and other pathways.^11^, ^12^, ^13^ For example, DBZ is used to treat chronic kidney disease by ameliorating fibroblast proliferation in the tubular epithelial cells via inhibition of the TGF‐β/Smad2/3 signalling pathway.^14^ Also, during the early stage of acute inflammation, DBZ can moderate the effects of acute colitis by protecting the goblet cells through inhibition of Notch signalling and reversing the down‐regulated mRNA of the goblet cell‐related genes *Math1* and *MUC2*.^12^ Finally, DBZ prevents AngII‐induced abdominal aortic aneurysm formation by inhibiting the accumulation of macrophages and CD4^+^ T cells, Th2 differentiation and ERK‐mediated angiogenesis.^13^ However, DBZ has not previously been studied in terms of promoting SC proliferation and HC regeneration in the inner ear.

In this study, we treated cultured neonatal mouse cochleae with DBZ to investigate its role in mammalian cochlear HC regeneration. We found that DBZ induced SC proliferation and promoted the mitotic regeneration of HCs with relatively mature stereocilia bundle structures by inhibiting Notch signalling and activating Wnt signalling. This study suggests that DBZ might be a new and effective therapeutic drug to promote the mitotic regeneration of HCs in the mammalian cochlea.

## 2 MATERIALS AND METHODS

### 2.1 Animals

Wild‐type neonatal (postnatal day (P)1) mice in the C57BL/6j background were from Fudan Medical School (Shanghai, China). Math1‐GFP transgenic mice were obtained from Dr Jane Johnson (University of Texas Southwestern Medical Center, Dallas).^15^ The care and use of animals was approved by the Institutional Animal Care and Use Committee of Fudan University in compliance with the NIH guidelines for the care and use of laboratory animals.

### 2.2 Organotypic culture of mouse cochleae

The mice were euthanized by carbon dioxide asphyxiation and decapitated, and their heads were placed in 75% ethanol and quickly transferred to chilled Hanks’ balanced salt solution (HBSS). The temporal bones were dissected out, the cochlea was isolated from the temporal bone using sterile procedures in ice‐cold HBSS, and the stria vascular and spiral ganglion were removed with fine forceps.

Explants of the organ of Corti were placed intact on polylysine‐coated cover glasses (Sigma, St. Louis, MO, USA) and maintained in four‐well culture dishes (Greiner Bio‐One, Frickenhausen, Germany) in culture medium composed of Dulbecco's modified Eagle's medium (DMEM) and F12 medium supplemented with N2 and B27 (Invitrogen/GIBCO/BRL, Carlsbad, CA) and 50 IU/mL penicillin (Sigma). The tissues were incubated at 37℃ in a humidified atmosphere at 95°C and 5% CO_2_.

### 2.3 Treatment of cultured cochleae

Cultures were switched to a culture medium with 10% FBS for about 2 hours to promote adherence to the wells and then treated with DBZ (GSI XX, (S,S)‐2‐[2‐(3,5‐Difluorophenyl)acetylamino]‐N‐(5‐methyl‐6‐oxo‐6,7‐dihydro‐5H‐dibenzo[b,d]azepin‐7‐yl)propionamide, CAS 209984‐56‐5; Calbiochem, Darmstadt, Germany) or with DAPT (γ‐secretase inhibitor IX, N‐[N‐(3,5‐difluorophenacetyl)‐l‐alanyl]‐S‐phenylglycinet‐butylester, EMD, Gibbstown, NJ) as a positive control for Notch inhibition. Both compounds were initially dissolved in sterile dimethylsulphoxide (DMSO, Sigma) and stored in aliquots at −20°C and then diluted to their final concentrations in culture medium immediately prior to use.

The explanted cochleae were treated with 5 μM or 10 μM DBZ for 3 days. Negative control cultures were incubated in 0.1% DMSO or PBS, while positive controls were incubated in 5 μM DAPT. The cell proliferation marker EdU (5‐ethynyl‐2'‐deoxyuridine, Invitrogen, Grand Island, NY), which is efficiently incorporated into newly synthesized DNA, was added to the culture medium at a concentration of 10 μM for the entire culture period.

Another group of explanted cochleae was treated with 1 mM neomycin (Sigma) for 12 hours and then thoroughly rinsed in fresh medium. These cochleae were then treated with 5 μM or 10 μM DBZ along with 10 μM EdU for 7 days.

### 2.4 Immunohistochemistry

The cultured cochleae were harvested and fixed for 30 minutes at room temperature with 4% paraformaldehyde in 0.1 M phosphate buffer (PBS) and then thoroughly rinsed with 0.01 M PBS. After being permeabilized in 0.5% Triton X‐100 in PBS (PBST) for 30 minutes at room temperature, the proliferating cells were labelled with EdU (Click‐iT EdU Alexa Fluor 488 imaging kit, Invitrogen, Carlsbad, CA, USA) for 30 minutes according to the manufacturer's protocol. The samples were then blocked with 10% horse serum in PBST for 30 minutes. The HCs were labelled with the primary antibodies at 4°C overnight, including rabbit antibody against Myo7a (1:500 dilution; Proteus Biosciences), goat polyclonal antibody against Sox2 (1:200 dilution; Santa Cruz Biotechnology), mouse antibody against Espin (1:200 dilution; BD Biosciences), mouse antibody against Parvalbumin (PAR) (1:500; Sigma‐Aldrich), rabbit antibody against Jagged‐1 (H‐114) (1:500 dilution; Santa Cruz Biotechnology) and chicken antibody against GFP (1:500 dilution; Abcam).

After being washed with PBST to remove the unbound primary antibodies, the samples were incubated for 1 hour at room temperature with the secondary antibodies donkey anti‐rabbit (1:500 dilution; Invitrogen), donkey anti‐goat (1:200 dilution; Invitrogen), donkey anti‐mouse (1:500 dilution; Invitrogen) and goat anti‐chicken (1:500 dilution, Invitrogen), all of which had been diluted in PBST, to visualize Myo7a, Sox2, Espin, Parvalbumin, Jagged‐1 and GFP, respectively. Then, the specimens were stained with 4’, 6‐diamidino‐2‐phenylindole dihydrochloride (DAPI) for 5 minutes at room temperature to visualize the cell nuclei.

### 2.5 Image acquisition and quantification

The fluorescence in the organ of Corti was visualized using a Nikon (Japan) Eclipse 80i microscope. The high‐magnification fluorescent images were obtained with a Leica TCS SP5 laser‐scanning microscope (Wetzlar, Germany). Cells were counted manually on the stored images using the ImageJ software (Wayne Rasband, NIH, USA). The whole‐mount cochleae were split into the apex, middle and base for counting the EdU‐labelled SCs and HCs, and the cell counts were obtained per 100 μm length of the cochlea. At least five samples in each group from three independent experiments were collected for statistical analysis. The cell counts for the control and treated groups were compared using Student's *t* test.

### 2.6 Scanning electron microscopy

The cultured cochleae in each group were rapidly placed into 2.5% glutaraldehyde (pH 7.4) in a refrigerator at 0‐4°C for at least 4 hours. The samples were washed with 0.1 M phosphate buffer and fixed with 1% osmium tetroxide for 2 hours. After further rinsing with phosphate buffer, the cochleae were dehydrated by gradient alcohol solutions and acetone and then soaked and embedded in acetone and embedding solution. The embedded tissues were put into an oven at 37°C overnight and then at 45°C for 12 hours and finally were polymerized at 60°C for 24 hours. Semi‐thin sections were prepared after staining, and then, ultra‐thin sections with a thickness of about 50‐60 nm were sliced on an LKB‐1 ultra‐thin slicer. After double staining with 3% uranyl acetate and lead citrate, the specimens were observed and photographed under a JEM‐1200EX transmission electron microscope.

### 2.7 RNA analysis

Ten cultured cochleae from independent culture groups were dissolved in RNALater for extraction of total RNA. RNA‐Seq libraries were generated using the Illumina mRNA‐Seq Sample Prep Kit. The RNA‐Seq data sets were processed by Salmon.^16^ The reference transcriptome sequence was downloaded from GENCODE [https://www.gencodegenes.org/mouse/] version M23. The tpm (transcripts per million) for each sample was imported into DESeq2^17^ to get a normalized expression matrix and differentially expressed gene list. The p‐value threshold for the comparison between DBZ‐treated and control samples was < 0.00001 with a logFC (log fold change) of > 2. Considering the smaller difference in HC regeneration or SC proliferation between DBZ and DAPT, the significance threshold was a p‐value of 0.05 and a logFC of 1.5. For the significance threshold for genes in gene sets, the threshold was set at a p‐value of 0.1 and a logFC of 1.25.

The gene set annotation was obtained from MSigDB [http://software.broadinstitute.org/gsea/msigdb/], and gene sets from the categories H, CP:BIOCARTA, CP:REACTOME, CP:KEGG, and BP were used for functional enrichment analysis using home‐made scripts. For each gene set, genes with a p‐value < 0.1 were considered significant (an additional logFC threshold of 1.5 was set for DBZ vs. Control).

Genes (either for DBZ vs. Control or for DBZ vs. DAPT) annotated by at least one of the four cilium‐related gene sets (“GO_CILIUM_MORPHOGENESIS”, “GO_CILIUM_MOVEMENT”, “GO_INNER_EAR_MORPHOGENESIS”, “GO_HAIR_CELL_DIFFERENTIATION”) and two signalling‐related gene sets (“KEGG_NOTCH_SIGNALING_PATHWAY”, “KEGG_WNT_SIGNALING_PATHWAY”) were entered into the STRING online server for network construction. The largest connected components of the networks were extracted and displayed with different shapes, colours and frame colours for each node indicating different gene features.

We also performed quantitative real‐time PCR (qRT‐PCR) to validate the differentially expressed genes. Three to five cultured cochleae from independent culture groups were pooled, and total RNA was extracted using the RNeasy Micro kit (QIAGEN) according to the manufacturer's protocol. The mRNA was reverse transcribed to synthesize cDNA using the GoScript Reverse Transcription System (Promega, Australia). qRT‐PCR was performed using GoTaq qPCR Master Mix (Promega, Australia). GAPDH was used for calibration. Each qRT‐PCR was performed in triplicate in a volume of 20 μL on a StepOne Plus Real‐Time PCR System (Applied Biosystems). Gene expression levels in the samples of each group were calculated using the comparative CT method where ΔΔCT = ΔCT sample ‐ ΔCT control and fold change = 2^−ΔΔCT^. Results are shown as mean ± SEM of three independent experiments, and Student's *t* test was used to assess statistical significance.

### 2.8 Statistics

All data were analysed with GraphPad Prism 6.0 using a two‐tailed, unpaired Student's *t* tests when comparing two groups or with a one‐way ANOVA followed by a Dunnett's multiple comparisons test when comparing more than two groups. All data are expressed as either a percentage or as the mean ± SEM. A *P*‐value < 0.05 was considered statistically significant.

## 3 RESULTS

### 3.1 DBZ induced the proliferation of SCs in neonatal mouse cochleae in vitro

**Figure 1:**
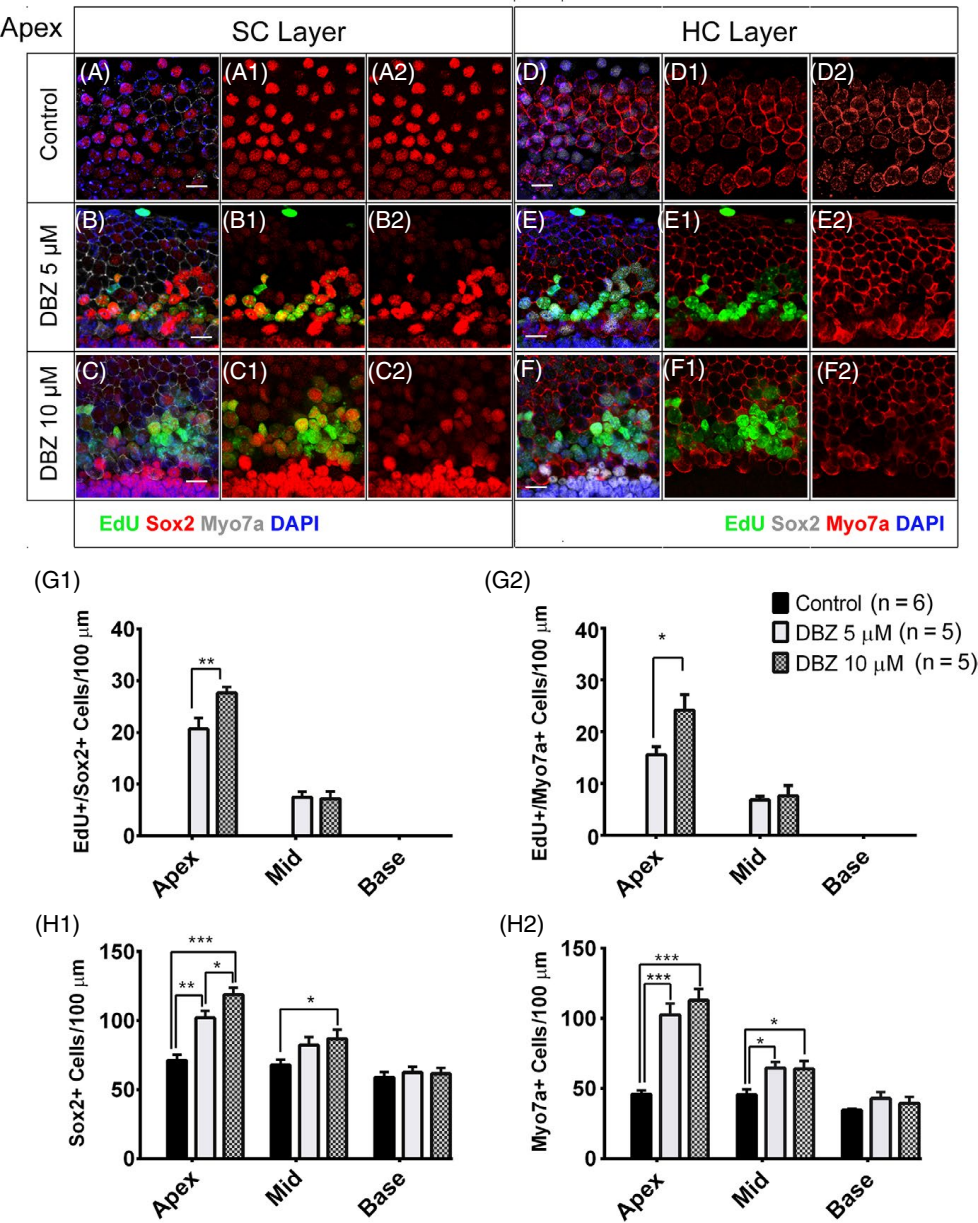
DBZ induced the proliferation of SCs, generated new HCs and increased the HC number in neonatal mouse cochleae in vitro. (Scale bar = 10 μm). (A‐A2) The neonatal mouse cochleae were cultured with EdU for 3 days, and there were no obvious EdU^+^/Sox2^+^ cells. (B‐B2) The cochleae were treated with 5 μM DBZ and EdU for 3 days, and there were some EdU^+^/Sox2^+^ cells, and these were mostly in the apical turn but also in the middle turn of the cochlea. The number of SCs increased compared to the control group, especially in the apical turn. (C‐C2) In the cochleae treated with 10 μM DBZ, there were many more EdU^+^/Sox2^+^ cells than in the 5 μM DBZ‐treated group. (D‐D2) The cochleae were cultured with EdU for 3 days, and there were no obvious EdU^+^/Myo7a^+^ cells. (E‐E2) The cochleae were treated with 5 μM DBZ and EdU for 3 days, and there were some EdU^+^/Myo7a^+^ cells in the apical and middle turns of the cochlea, and most of the EdU^+^/Myo7a^+^ cells were in the apical turn. (F‐F2) In the cochleae treated with 10 μM DBZ, there were many more EdU^+^/Myo7a^+^ cells than in the 5 μM DBZ‐treated group. Histograms show the numbers of EdU^+^/Sox2^+^ cells (G1), Sox2^+^ cells (H1), EdU^+^/Myo7a^+^ cells (G2) and Myo7a^+^ cells (H2) in the control and DBZ‐treated cochleae. Most of the EdU^+^/Sox2^+^ cells and EdU^+^/Myo7a^+^ cells were in the apical turn of the cochlea (*P < .05, **P < .01, ***P < .001)

We first investigated the effect of DBZ on promoting the proliferation of SCs in cultured mouse cochleae. Cochleae were harvested from P1 C57/BL6 mice and then cultured in DMEM/F12 media with 5 μM or 10 μM DBZ for 3 days, and 10 μM EdU was added for the entire culture period (Figure 1, Figure S1). No Sox2^+^/EdU^+^ cells were observed in the vehicle control group (Figure 1A, Figure S1A, S2A). There were some proliferating SCs as indicated by Sox2^+^/EdU^+^ cells in the cochleae treated with 5 μM DBZ, and these were primarily seen in the apical turn with some in the middle turn of the cochlea, while very few were seen in the basal turn (Figure 1B, 1G1, Figure S1B, S2B). In the cochleae treated with 10 μM DBZ, there were significantly more Sox2^+^/EdU^+^ SCs in the apical and middle turns, with most of the Sox2^+^/EdU^+^ SCs still seen in the apical turn (Figure 1C, G1, Figure S1C, S2C). The total number of SCs increased after the DBZ treatment (Figure 1H1).

### 3.2 DBZ generated new HCs and increased the HC number in neonatal mouse cochleae in vitro

To investigate the effect of DBZ in HCs in cultured mouse cochleae, we cultured P1 mouse cochleae with 5 μM or 10 μM DBZ and 10 μM EdU for 3 days (Figure 1, Figure S1, S2). In the control group, there were no obvious Myo7a^+^/EdU^+^ cells (Figure 1D, Figure S1D, S2A). The total HC number was increased, and there were some Myo7a^+^/EdU^+^ cells in the cochleae treated with 5 μM DBZ, and these were mostly in the apical turn but also in the middle turn of the cochlea, while there were no obvious Myo7a^+^/EdU^+^ cells in the basal turn (Figure 1E, 1 G2, Figure S1E, S2B). In the cochleae treated with 10 μM DBZ, the total HC number was significantly increased and there were many more Myo7a^+^/EdU^+^ cells in the apical and middle turns of the cochleae (Figure 1F, G2, Figure S1F, S2C).

Interestingly, among the Myo7a^+^/EdU^+^ cells in the DBZ‐treated cochleae, most of them were also Myo7a^+^/EdU^+^/Sox2^+^ triple positive, and only a few of the Myo7a^+^/EdU^+^ cells were not Sox2^+^, suggesting that the newly regenerated HCs originated from the proliferating Sox2^+^ SCs (Figure 1D,F, Figure S1D, S1F). Together, these results demonstrate that DBZ treatment increases the HC number and promotes supernumerary HCs in the apical and middle turns of cultured cochleae in vitro (Figure 1H2).

### 3.3 The newly generated HCs induced by DBZ had normal stereocilia bundle structures

**Figure 2:**
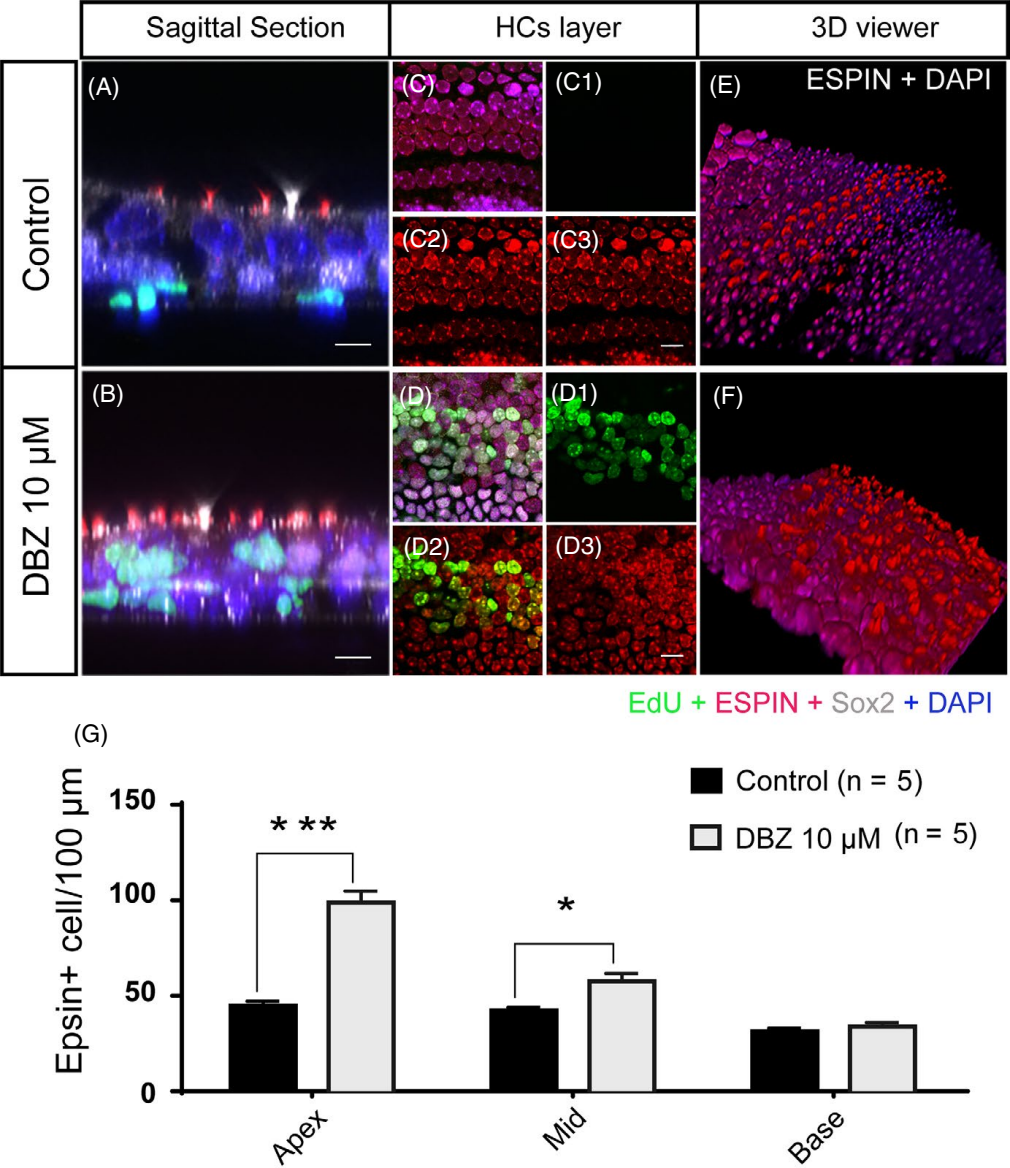
The newly generated HCs induced by DBZ had stereocilia bundle structures. (Scale bar = 10 μm). (A, C, E) The control cochleae were cultured with EdU for 3 days, and there were no obvious EdU^+^ cells. The sagittal plane of the Espin‐stained cochlea shows that the HCs had stereocilia bundles. (B, D, F) The cochleae were cultured with 10 μM DBZ and EdU for 3 days, and there were many EdU^+^ cells in the HC region. The sagittal plane of the Espin‐stained cochlea shows that the EdU^+^ cells had more stereocilia bundles than controls. The 3D stereograms of Espin staining of HCs in the control group (E) and DBZ‐treated group (F) showing that the number of HCs with stereocilia bundle structures increased in the DBZ‐treated group. (G) The histogram shows the numbers of Espin^+^ cells in the control and DBZ‐treated cochleae. The number of Espin^+^ cells in DBZ‐treated group increased compared to the control group, especially in the apical turn. (*P < .05, ***P < .001)

The P1 mouse cochleae were treated with 10 μM DBZ and 10 μM EdU for 3 days and then stained with the antibody against Espin, which is an actin binding and bundling protein known to participate in stereocilia elongation during development.^18^ The majority of the DBZ‐induced EdU^+^/Sox2^+^ cells also showed positive Espin staining. There were many Espin^+^/EdU^+^ cells in the apical and middle turns of the cochleae, and the number of Espin^+^ cells was clearly increased (Figure 2B,D‐D3, 2F) compared to the control group (Figure 2A,C‐C3,E). These results showed that the newly generated HCs induced by DBZ had stereocilia bundle structures. The numbers of Espin + cells significantly increased in the DBZ‐treated group, especially in the apical and middle turns (Figure 2G).

### 3.4 DBZ treatment reduced the expression of Jagged‐1 on the cytomembrane of SCs

**Figure 3:**
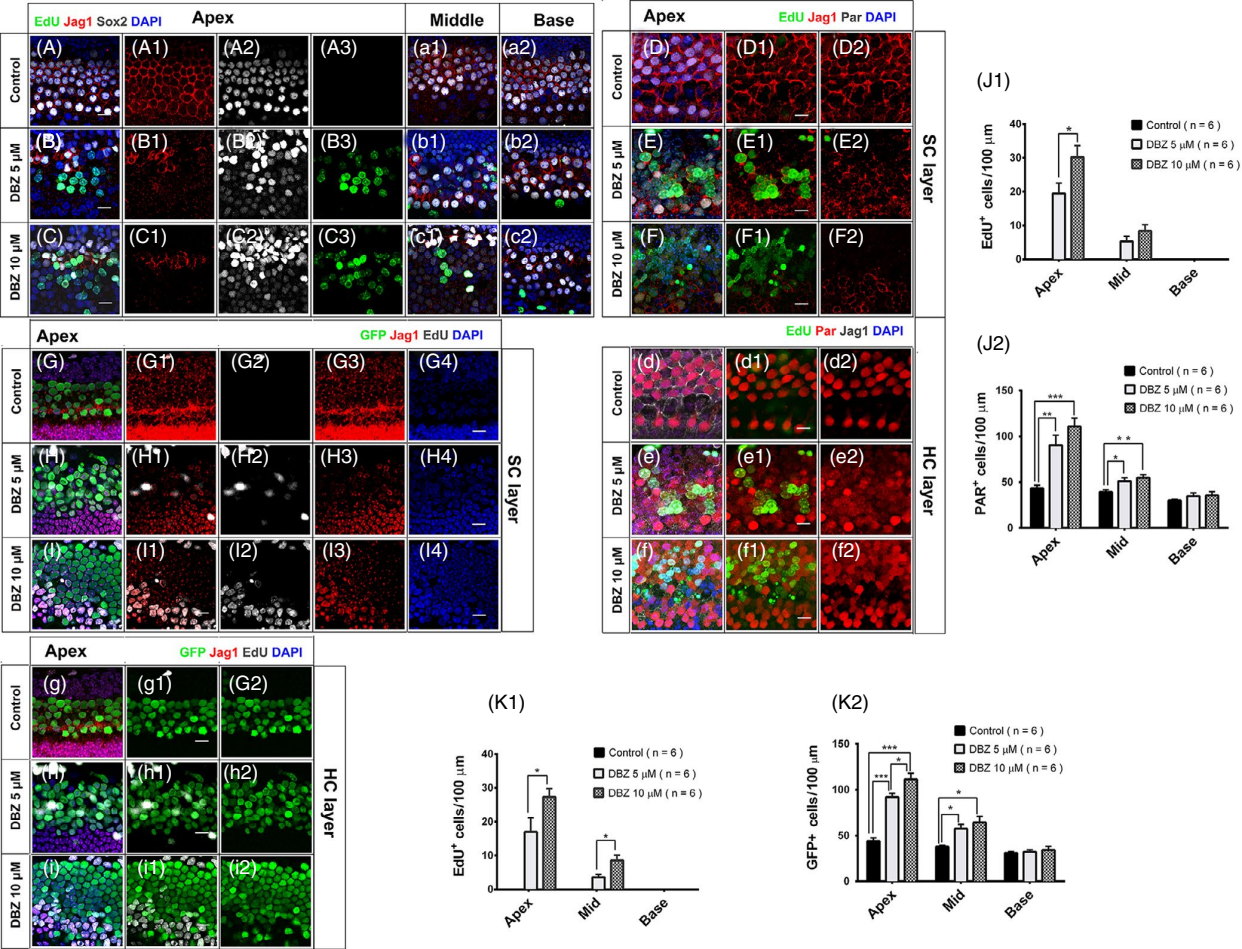
DBZ reduced the expression of Jagged‐1 on the cytomembrane of SCs in a region and dose‐dependent manner (Scale bar = 10 μm). (A‐A3, a1‐a2) The cochleae were cultured with EdU for 3 days. There was obvious Jagged‐1 protein expression on the cytomembrane of the SCs. (B‐B3, b1‐b2) The cochleae were treated with 5 μM DBZ and EdU for 3 days, and the expression of Jagged‐1 decreased compared to the control group, especially in the apical and middle turns of the cochlea. There were some EdU^+^/Sox2^+^ cells in the apical turn of the cochlea, and the number of Sox2^+^ cells increased. (C‐C3, c1‐c2) In the 10 μM DBZ‐treated group, the expression of Jagged‐1 decreased in all three turns of the cochlea. There were some EdU^+^/Sox2^+^ cells in the apical and middle turns of the cochlea, and the number of Sox2^+^ cells increased. (D‐D2, d‐d2) In the control group, staining of Jagged‐1 showed obvious expression in the apical turn of the cochlea. There were no obvious EdU^+^ cells in the HC region. (E‐E2, e‐e2) In the 5 μM DBZ‐treated group, there were some EdU^+^/Sox2^+^ cells in the apical turn of the cochlea. The expression of Jagged‐1 decreased compared to the control group in the apical turn of the cochlea. (e) There were some EdU^+^/Parvalbumin^+^ cells in the apical turn of the cochlea. (F‐F2, f‐f2) In the 10 μM DBZ‐treated group, there were many more EdU^+^/Sox2^+^ cells in the apical turn of the cochleae than in the 5 μM DBZ‐treated group. (f) There were many more EdU^+^/Parvalbumin^+^ cells in the apical turn of cochleae than in the 5 μM DBZ‐treated group. Histograms show the numbers of EdU^+^ cells (J1) and Parvalbumin^+^ cells (J2) in the control and DBZ‐treated cochleae. Most of the EdU^+^ cells were in the apical turn of the cochlea. The number of Parvalbumin^+^ cells in the DBZ‐treated group increased compared to the control group, especially in the apical turn (*P < .05, **P < .01, ***P < .001). (G‐G4, g‐g2) In the control Math1‐GFP transgenic mice, there were no obvious EdU^+^ cells in the HC region, and Jagged‐1 staining was normal in the apical turn of the cochlea. (H‐H4, h‐h2) In the 5 μM DBZ‐treated Math1‐GFP transgenic mice, there were some EdU^+^/GFP^+^ cells in the apical turn of the cochlea. The expression of Jagged‐1 was weak and decreased compared to the control group in the apical turn of the cochlea, while the number of HCs increased compared to the control group. (I‐I4, i‐i2) In the 10 μM DBZ‐treated Math1 mice, there were many more EdU^+^/GFP^+^ cells in the apical turn of the cochleae compared to the 5 μM DBZ‐treated group. Histograms show the numbers of EdU^+^ cells (K1) and GFP^+^ cells (K2) in the control and DBZ‐treated cochleae. Most of the EdU^+^ cells were in the apical turn of cochlea. The number of GFP^+^ cells in DBZ‐treated group increased compared to the control group, especially in the apical turn (*P < .05, **P < .01, ***P < .001)

Previous reports have shown that Notch inhibition can promote SC proliferation and subsequent differentiation into HCs^4^, ^19^, ^20^ and that DBZ treatment can inhibit Notch signalling in the intestinal tract and renal tissue and in many other tissues.^14^, ^21^ To validate that DBZ treatment inhibits Notch signalling in the cochlea, the cultured P1 cochleae were treated with 5 μM or 10 μM DBZ and 10 μM EdU for 3 days, and then the cochleae were stained with antibodies against Jagged‐1, which is one of the ligands for the Notch signalling pathway, the SC marker Sox2, and the HC marker Parvalbumin, which was demonstrated to be an early marker of non‐mitotically generated HCs^22^ (Figure 3). In the control group, the Sox2^+^ SCs were well organized in the cochlea and there was obvious Jagged‐1 protein expression on the cytomembrane of the SCs (Figure 3A‐A3,a1‐a2,D‐D2,d‐d2). In the 5 μM DBZ‐treated group, the Jagged‐1 protein expression on the cytomembrane of SCs was significantly decreased in the apical and middle turns of the cochlea compared to controls, while the Jagged‐1 expression in the basal turn remained unchanged compared to the controls (Figure 3B‐B3,b1‐b2,E‐E2,e‐e2), which was consistent with our results above showing that DBZ treatment increased SC proliferation and differentiation into HCs in the apical and middle turns of the cochlea, but not in the basal turn (Figure 3J1‐J2). In the 10 μM DBZ‐treated group, the expression of Jagged‐1 was almost undetectable in the apical turn of the cochlea (Figure 3C‐C3, c1‐c2, F‐F2, f‐f2). Together, these results demonstrated that DBZ treatment significantly reduces the expression of Jagged‐1 on the cytomembrane of SCs of the cochlea in a dose‐dependent manner, which might be one of the mechanisms through which DBZ treatment can induce SC proliferation and differentiation into HCs.

To further confirm that DBZ can induce sensory cell proliferation and HC differentiation, we cultured Math1‐GFP transgenic mouse cochleae with DBZ for 72 hours and then stained the cochleae for GFP, Jagged‐1 and EdU. As previously reported, the expression of Math1 is limited to HCs at an early stage of development, and Math1‐GFP can be used as a marker for HC differentiation or for immature HCs.^15^ DBZ induced SC proliferation and supernumerary HC generation in the apical and middle turns of the Math1 mouse cochlea, especially in the apical turn (Figure 3H‐H4, I‐I2). This effect was more obvious in the 10 μM DBZ‐treated group compared to the 5 μM DBZ‐treated group (Figure 3H‐H4,h‐h2,I‐I4,i‐i2,K1‐K2). In the Math1 mouse cochlea, DBZ also decreased Jagged‐1 expression. In the control group, we could observe Jagged‐1 in the apical turn of the Math1 mouse cochlea (Figure 3G‐G4,g‐g2). In the 5 μM DBZ‐treated group, there was no obvious Jagged‐1 staining in the apical or middle turns of the cochlea, and only a little Jagged‐1 staining was seen in the basal turn (Figure 3H‐H4, h‐h2). In the 10 μM DBZ‐treated group, there was no obvious Jagged‐1 staining in the apical turn of the cochlea (Figure 3I‐I4,i‐i2).

### 3.5 DBZ induced the proliferation of SCs and mitotic regeneration of HCs after HC ablation in neonatal mouse cochleae in vitro

**Figure 4:**
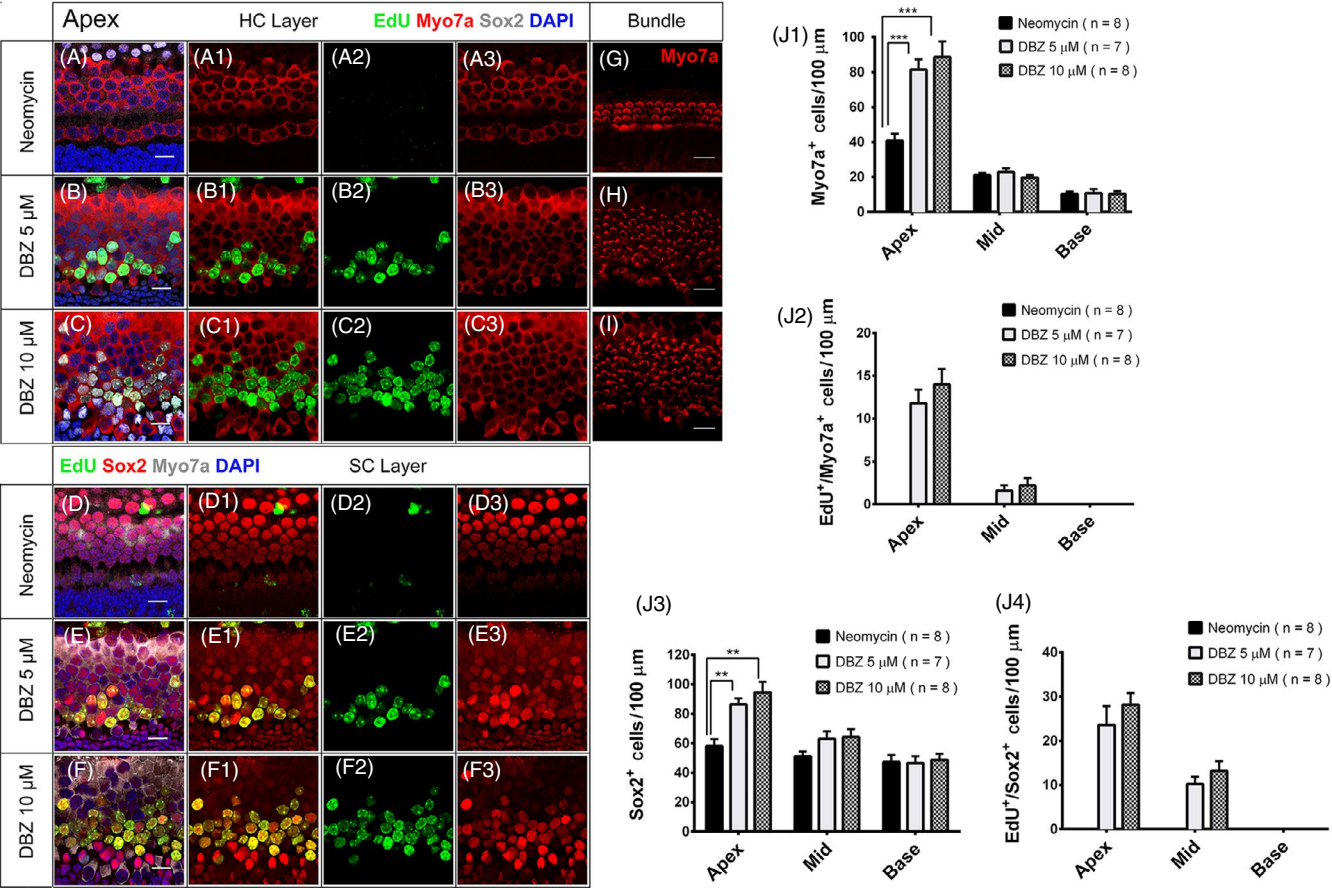
DBZ induced SC proliferation and HC regeneration after HC ablation in neonatal mouse cochleae in vitro. The newly regenerated HCs had stereocilia bundle structures. (Scale bar = 10 μm). (A‐A3) The neonatal mouse cochleae were treated with 1.0 mM neomycin for 12 hours and then cultured with EdU for 3 days, and there were no obvious EdU^+^ cells in the HC region. (B‐B3) The cochleae were treated with 1.0 mM neomycin for 12 hours and then treated with 5 μM DBZ and EdU for 3 days, and there were some EdU^+^/Myo7a^+^ cells in the apical turn of the cochlea. The number of HCs increased compared to the control group, especially in the apical turn. (C‐C3) In the cochleae treated with 1.0 mM neomycin and then with 10 μM DBZ and EdU there were many more EdU^+^/Myo7a^+^ cells compared to the 5 μM DBZ‐treated group. (D‐D3) The neonatal mouse cochleae were treated with 1.0 mM neomycin and then cultured with EdU for 3 days, and there were no obvious EdU^+^/Sox2^+^ cells in the HC region. (E‐E3) The cochleae were treated with 1.0 mM neomycin for 12 hours and then treated with 5 μM DBZ and EdU for 3 days, and there were some EdU^+^/Sox2^+^ cells in the apical turn of the cochleae. (F‐F3) In the cochleae treated with 1.0 mM neomycin and then with 10 μM DBZ and EdU, there were many more EdU^+^/Sox2^+^ cells in the apical turn compared to the 5 μM DBZ‐treated cochleae. (G‐I) The number of HC stereocilia bundles increased with the DBZ concentration. (Scale bar = 10 μm). Histograms show the numbers of Myo7a^+^ cells (J1), EdU^+^/Myo7a^+^ cells (J2), Sox2^+^ cells (J3) and EdU^+^/Sox2^+^ cells (J4) in the control and DBZ‐treated cochleae. Most of the EdU^+^/Myo7a^+^ cells and EdU^+^/Sox2^+^ cells were in the apical turn of the cochlea. The number of Myo7a^+^ cells and Sox2^+^ cells in DBZ‐treated group increased compared to the control group, especially in the apical turn (*P < .05, **P < .01, ***P < .001)

Neomycin is widely used to induce the HC damage model. To investigate the detailed effect of DBZ in promoting the proliferation of SCs and the regeneration of HCs after HC injury in cultured mouse cochleae, 1.0 mM neomycin was added to the DMEM/F12 media for 12 hours followed by 5 μM or 10 μM DBZ with EdU for 7 days (Figure 4). No Myo7a^+^/EdU^+^ HCs were observed in the vehicle control group (Figure 4A‐A3,J1‐J2), while large numbers of Myo7a^+^/EdU^+^ HCs were observed in the apical and middle turns of the 5 μM and 10 μM DBZ‐treated cochleae (Figure 4B‐B3,C‐C3,J1‐J2). There were no significant numbers of Myo7a^+^/EdU^+^ HCs in the basal turns in DBZ‐treated cochleae. This was consistent with the results in undamaged neonatal cochleae, and among the Myo7a^+^/EdU^+^ cells in the DBZ‐treated cochleae, most of them were also Myo7a^+^/EdU^+^/Sox2^+^ triple positive and only a few of the Myo7a^+^/EdU^+^ cells were not Sox2^+^, suggesting that the mitotically regenerated HCs originated from the proliferated Sox2^+^ SCs (Figure 4B‐B3,C‐C3, J3). Also, no Sox2^+^/EdU^+^ cells were observed in the vehicle control group (Figure 4D‐D3,J4), while large numbers of Sox2^+^/EdU^+^ SCs were observed in the apical and middle turns of the 5 μM and 10 μM DBZ‐treated cochleae (Figure 4E‐E3,F‐F3,J4). There were no obvious Sox2^+^/EdU^+^ SCs in the basal turns in the control or DBZ‐treated cochleae.

These results demonstrate that DBZ treatment increases the HC and SC number by promoting SC proliferation and mitotic HC regeneration in the apical and middle turns of cultured cochleae after HC injury in vitro.

### 3.6 The newly regenerated HCs induced by DBZ treatment had HC stereocilia bundle structures

To determine whether the newly regenerated HCs also had stereocilia bundles, P1 mouse cochleae were treated with 1.0 mM neomycin for 12 hours and then cultured in DMEM/F12 media with 5 μM DBZ or 10 μM DBZ and 10 μM EdU for 7 days (Figure 4, Figure S2). DBZ induced many Myo7a^+^/EdU^+^ cells and Sox2^+^/EdU^+^ cells in the apical turn of the cochlea (Figure 4B‐B3,C‐C3), and the newly regenerated HCs had hair bundles (Figure 4G,H,I, Figure S3). These results showed that most of the newly regenerated HCs induced by DBZ had HC stereocilia bundle structures.

### 3.7 DBZ‐induced newly regenerated HCs had more mature bundle structures than those induced by DAPT in the apical turn of the cochlea

**Figure 5:**
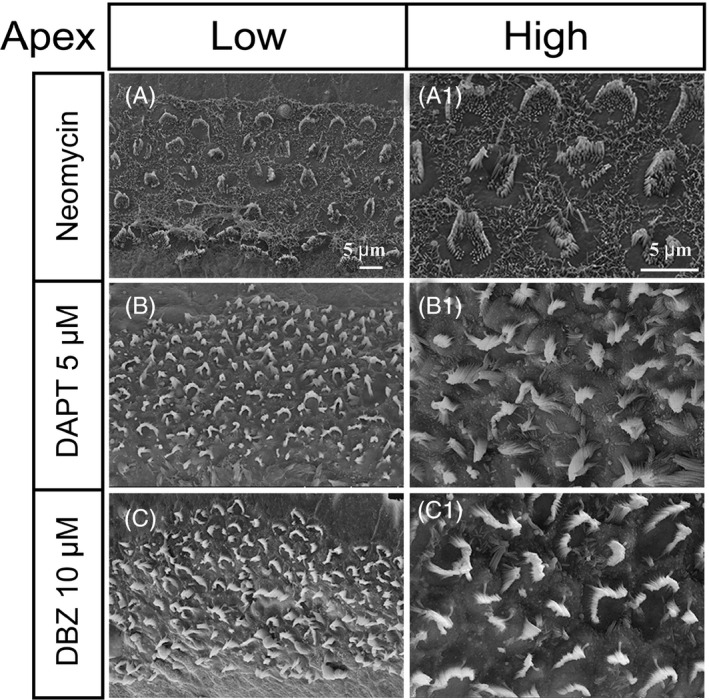
The HCs regenerated by DBZ treatment had more mature stereocilia bundles compared to those regenerated by DAPT treatment. (A‐A1) In the neomycin‐treated group, some of the HCs were absent and the bundles of the surviving HCs were arranged in abnormal structures. (B‐B1) In the 5 μM DAPT‐treated group, the newly regenerated HCs had short and disordered immature bundle structures in the apical turn of the cochlea. (C‐C1) In the 10 μM DBZ‐treated group, the newly regenerated HCs had longer and more organized mature bundle structures than the DAPT‐treated group

To investigate the bundle structure of newly regenerated HCs induced by DBZ, we cultured P1 mouse cochleae and treated them with 1.0 mM neomycin for 12 hours, then treated them with 5 μM DAPT or 10 μM DBZ and 10 μM EdU for 7 days. Electron microscope scanning showed that in the controls neomycin induced abnormal bundle structures (Figure 5A‐A1), while in the 5 μM DAPT‐treated group the newly regenerated HCs in the apical turn of the cochlea had shorter and disordered immature bundle structures (Figure 5B‐B1) and in the 10 μM DBZ‐treated group the new HCs had longer and more organized mature bundle structures (Figure 5C‐C1).

### 3.8 The mechanism of DBZ‐induced SC proliferation and HC regeneration

**Figure 6:**
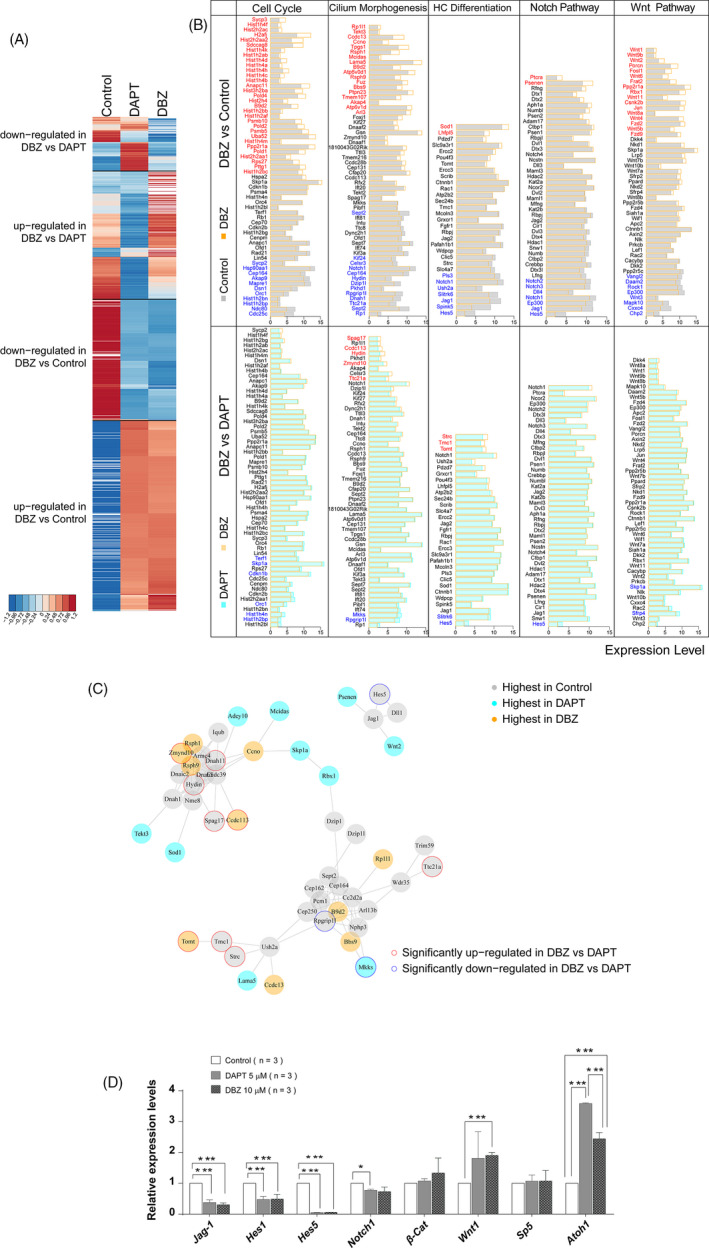
Gene expression and related mechanisms involved in DBZ‐induced HC regeneration. (A) Hierarchical clustering of expression patterns of all differentially expressed genes. Red represents up‐regulated expression levels, and blue represents down‐regulated expression levels. Each row represents one gene, and each column represents one experimental group. (B) Differentially expressed genes in the DBZ vs. control group and the DBZ vs. DAPT group, including genes involved in the cell cycle, cell cilium morphogenesis, HC differentiation and Notch/Wnt signalling pathways. Significantly up‐regulated genes are labelled with red, and down‐regulated genes are labelled with blue. The significance between DBZ vs. control was set at logFC > 1.25 and p‐value < 0.1. The threshold between DBZ vs. DAPT was set at p‐value < 0.1. If the number of genes involved in each gene set was more than 60, the most significantly differentiated 60 genes are shown (ordered by logFC value). (C) Protein interaction network analysis of genes involved in cilium‐related functions that are significantly up‐regulated (red) and down‐regulated (blue) in the control, DAPT and DBZ‐treated groups. The grey lines indicate protein‐protein interactions recorded in the STRING database. (D) The results of qRT‐PCR showed that the DBZ treatment led to the decreased expression of *Jagged‐1*, *Hes1*, *Hes5*, *Notch1* and increased expression of *β‐catenin*, *Wnt1* and *Math1*, which was consistent with the RNA‐seq results

To assess the genome‐wide gene expression among the neomycin‐only controls, the DBZ‐treated group and the DAPT‐treated group, we compared the transcripts of the cochleae from each group using mRNA sequencing. We selected the significantly differentially expressed genes between the DBZ‐treated groups and the DAPT‐treated and control groups. Their expression patterns are shown in Figure 6A with detailed pathway information and related mechanisms shown in Figure 6B. Among the differentially expressed genes between the DBZ and control groups (Table S1), we found that many of the his genes involved in cell cycle progression were significantly up‐regulated. *Sod1*—which is involved in HC differentiation—was up‐regulated and *Pitx2* and *Hes5* were down‐regulated; the *Wnt2*, *Wnt1* and *Wnt6* genes in the Wnt pathway were up‐regulated; and the *Jag1*, *Notch1*, *Notch2* and *Notch3* genes were down‐regulated. These results further confirmed that DBZ induced SC proliferation and HC differentiation by inhibiting the Notch signalling pathway and by activating the Wnt signalling pathway. Among the differentially expressed genes between the DBZ‐treated and the DAPT‐treated groups, the *Hes5*, *Hes1* and *Jag1* genes were down‐regulated. This indicated that DBZ inhibited the Notch signalling pathway to a greater extent than DAPT.

In order to get a comprehensive view of the gene network involved in HC regeneration, we performed a STRING protein‐protein interaction analysis (Figure 6C), which was used to assemble the predicted networks of the differentially expressed genes with the functional categories that are involved in inner ear HC development and are predicted to participate in SC proliferation and HC regeneration. Among the genes involved in HC and stereocilia regeneration, *Spag17*, *Hydin, Dnah11*, *Zmynd10*, *Ccdc113*, *Tomt*, *Strc*, *Tmc1* and *Ttc21a* were significantly up‐regulated, while *Skp1a*, *Rpgrip1l*, *Mkks* and *Hes5* were significantly down‐regulated in the DBZ‐treated group compared to the DAPT‐treated group.

The qRT‐PCR experiment results also showed in the DBZ‐ or DAPT‐treated groups, the *Jag1*, *Hes1*, *Hes5* and *Notch1* genes were down‐regulated, and the *β‐catenin*, *Wnt1* and *Atoh1* genes up‐regulated (Figure 6D). These results were consisted with the RNA‐seq, which also indicated that DBZ inhibited the Notch signalling pathway and activating the Wnt signalling pathway.

## 4 DISCUSSION

Mature mammalian cochlear HCs do not spontaneously mount a proliferative response after HC degeneration compared to the avian inner ear and the zebrafish lateral line^23^, ^24^ in which new HCs are generated from the surrounding SCs by direct transdifferentiation or by mitotic regeneration.^25^, ^26^, ^27^ In this study, adding DBZ to the cultured neonatal mouse cochlea induced SC proliferation and HC regeneration. Among the EdU^+^ cells in the DBZ‐treated cochleae, there were EdU^+^/Myo7a^+^ and EdU^+^/Sox2^+^ double‐positive cells as well as EdU^+^/Myo7a^+^/Sox2^+^ triple‐positive cells. There were many more EdU^+^/Myo7a^+^/Sox2^+^ triple‐positive cells, and only a few cells were EdU^+^/Myo7a^+^/Sox2^−^, which means that the new HCs were mostly derived from SC proliferation and transdifferentiation rather than from proliferation of HCs themselves. We also observed that most of the proliferating SCs arose from the base layer of the cochlea into the surface layer surrounding the HCs, where some of the SCs then transdifferentiated into new HCs.

Our research suggests that DBZ administration results in decreased expression of the Notch ligand Jagged‐1 and the Notch target genes *Hes1*, *Hes5*, *Notch1*, *Notch2* and *Notch3* in the wild‐type neonatal mice, while in the Math1‐GFP neonatal transgenic mice DBZ treatment induced new HCs that were Math1‐GFP positive and had decreased expression of Jagged‐1. Together these results suggest that DBZ induces SC proliferation and HC regeneration by inhibiting Notch signalling. Several γ‐secretase inhibitors have been developed recently that can block the Notch signalling pathway, and these are of considerable interest because of their potential use in many diseases. The therapeutic potential of γ‐secretase inhibitors is based on the Notch signalling pathway being essential in controlling cell proliferation and differentiation,^21^, ^28^ and many previous studies have shown that inhibiting the Notch pathway by various methods can regenerate new cochlear HCs.^29^, ^30^, ^31^

The proliferation of SCs and generation of new HCs in the neonatal mouse cochlea induced by DBZ inhibition of the Notch signalling pathway was more obvious in the apical turn of the cochlea than in the middle and basal turns. Thus, the apical turn maintains its capacity for proliferation and trans‐differentiation of SCs and HCs for a longer time compared to the middle and basal turns. This result was similar to what has been reported in previous studies.^19^, ^32^, ^33^ The induced SC proliferation and HC regeneration was more obvious with the larger dose of DBZ, and together these results show that DBZ promotes the proliferation of SCs and the regeneration of HCs in a region and dose‐dependent manner.

We show here that the newly regenerated HCs induced by DBZ treatment had stereocilia bundle structures, which showed that DBZ has a distinct advantage over many other γ‐secretase inhibitors reported in a previous study.^18^ The stereocilia bundle is the site of mechanoelectrical transduction of HCs and is essential for HCs to maintain their physiological function.^18^, ^34^ The fact that DBZ promotes the regeneration of HCs with intact stereocilia bundles is a favourable indication that DBZ might lead to recovery of hearing function.

We performed RNA‐seq and analysed the gene expression between the control group, the DBZ‐treated group and the DAPT‐treated group in order to further explore the mechanism behind the SC proliferation and HC regeneration induced by DBZ treatment. Among the differentially expressed genes between the control and DBZ‐treated group, we found that the Notch target genes *Jag1*, *Hes1*, *Hes5*, *Notch1*, *Notch2* and *Notch3* were down‐regulated while the mRNA expression of the *Wnt2*, *Wnt1* and *Wnt6* genes was increased, which suggests that the effect of DBZ on SC proliferation and HC regeneration is mediated through inhibition of the Notch signalling pathway and activation of the Wnt signalling pathway. In the differentially expressed genes between the DBZ and the DAPT‐treated groups, the Notch‐related genes *Hes5*, *Hes1* and *Jag1* were down‐regulated, which indicated that DBZ inhibited the Notch signalling pathway more so than DAPT. In addition, the cilia‐related genes *Hydin*, *Dnah11*, *Zmynd10*, *Ccdc113*, *Strc* and *Ttc21a* were significantly up‐regulated in the DBZ‐treated group, suggesting that DBZ might induce the regeneration of new HCs with relatively mature stereocilia bundles.

In summary, DBZ can induce the proliferation of SCs and promote the mitotic regeneration of HCs with mature stereocilia bundles in both the absence and presence of HC injury in the mouse cochlea and in a dose‐dependent manner. DBZ treatment can significantly down‐regulate Notch signalling genes and activate Wnt signalling and some cilia‐related genes in the mouse cochlea, which is likely the mechanism through which DBZ promotes SC proliferation and differentiation into HCs with relatively mature stereocilia. Thus, DBZ might be a new and useful therapeutic drug for HC regeneration in the mammalian cochlea to treat hearing loss.

## CONFLICT OF INTEREST

The authors declare that they have no competing interests.

## AUTHOR CONTRIBUTION

HW Li and S Sun involved in study design. JF Wu, W Li, L Zhou and LP Zhao involved in data collection. S Sun and HW Li involved in contribution of reagent and materials. XR Dong involved in contribution of analytical tools. JF Wu and S Sun involved in data analysis. JF Wu, S Sun and HW Li involved in manuscript preparation.

## Supporting information

Fig S1

Fig S2

Fig S3

Table S1

Supplementary Material

## Data Availability

The data that support the findings of this study are available from the corresponding author upon reasonable request.
