# Supplementary figures and images for: Dibenzazepine promotes cochlear supporting cell proliferation and hair cell regeneration in neonatal mice
Source: Cell Prolif. 2020 Jul 17;53(9):e12872. doi: 10.1111/cpr.12872 (PMC7507434; doi:10.1111/cpr.12872)

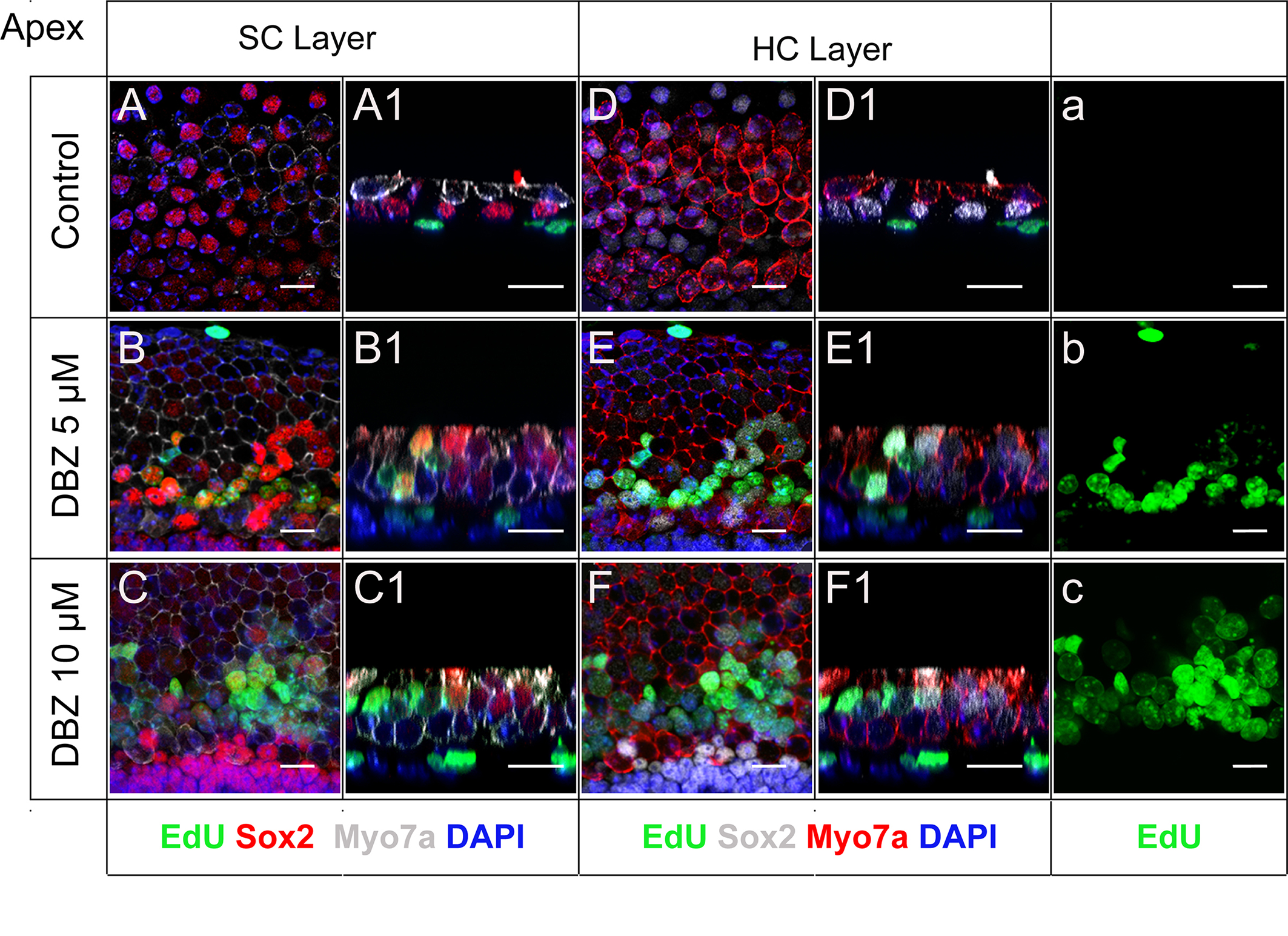

Supplement: Supplementary file 1 — Fig S1 [file CPR-53-e12872-s001.tif]

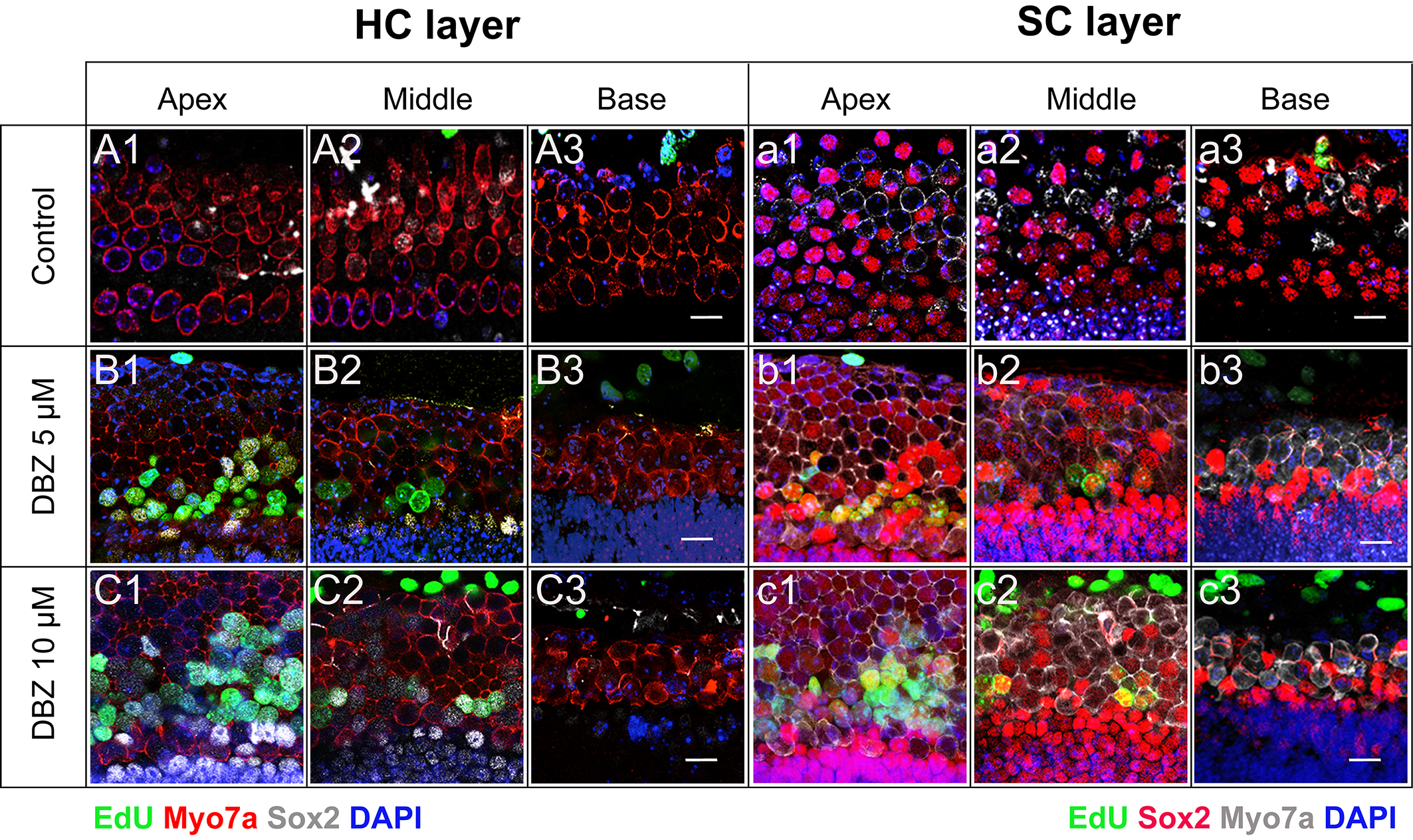

Supplement: Supplementary file 2 — Fig S2 [file CPR-53-e12872-s002.tif]

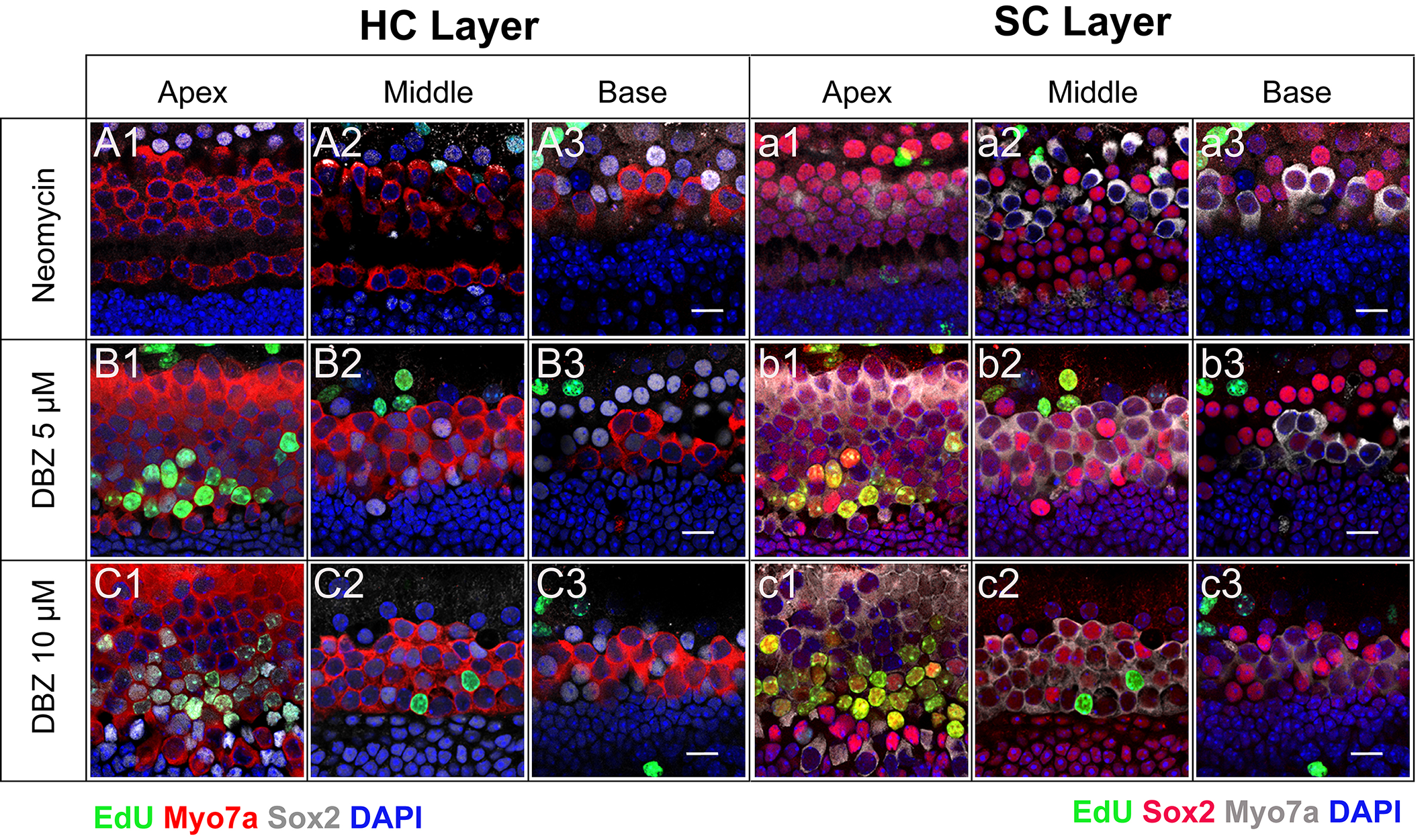

Supplement: Supplementary file 3 — Fig S3 [file CPR-53-e12872-s003.tif]
