# Supplementary material for: Dibenzazepine promotes cochlear supporting cell proliferation and hair cell regeneration in neonatal mice
Source: Cell Prolif. 2020 Jul 17;53(9):e12872. doi: 10.1111/cpr.12872 (PMC7507434; doi:10.1111/cpr.12872)
Supplement: Supplementary file 5 — Supplementary Material [file CPR-53-e12872-s005.docx]

**Supplementary Figure legends:**

**Figure S1**: **DBZ increases the SC proliferation and promotes supernumerary HCs in neonatal mouse cochleae in vitro.** (Scale bar = 10 μm)

**Figure S2：DBZ increases SC proliferation and promotes mitotic HC regeneration mostly in the apical turn followed by the middle turn of the cochlea, while very few HCs are regenerated in the basal turn of the cochlea.** (Scale bar = 10 μm)

HC layer (A1-C3): The apical turn (A1, B1, C1), the middle turn (A2, B2, C2), and the basal turn (A3, B3, C3). (A1-A3) The control group, cultured with media only; (B1-B3) Samples cultured with 5 μM DBZ; and (C1-C3) Samples cultured with 10 μM DBZ.

SC layer (a1-c3): The apical turn (a1, b1, c1), the middle turn (a2, b2, c2) and the basal turn (a3, b3, c3). (a1-a3) The control group, cultured with media only; (b1-b3) Samples cultured with 5 μM DBZ; and (c1-c3) Samples cultured with 10 μM DBZ.

**Figure S3: DBZ increases SC proliferation and HC regeneration after HC ablation in the cochleae mostly in the apical turn followed by the middle turn, while very few HCs are regenerated in the basal turn of the cochleae.** (Scale bar = 10 μm)

HC layer (A1-C3): The apical turn (A1, B1, C1), the middle turn (A2, B2, C2) and the basal turn (A3, B3, C3). (A1-A3) The neomycin control group, cultured with neomycin only; (B1-B3) Samples cultured with neomycin and 5 μM DBZ; and (C1-C3) Samples cultured with neomycin and 10 μM DBZ.

SC layer (a1-c3): The apical turn (a1, b1, c1), the middle turn (a2, b2, c2) and the basal turn (a3, b3, c3). (a1-a3) The neomycin control group, cultured with neomycin only; (b1-b3) Samples cultured with neomycin and 5 μM DBZ; and (c1-c3) Samples cultured with neomycin and 10 μM DBZ.
